# *Triatoma dimidiata,* domestic animals and acute Chagas disease: a 10-year follow-up after an eco-bio-social intervention

**DOI:** 10.1186/s13071-025-06897-7

**Published:** 2025-07-04

**Authors:** Jose G. Juarez, Andrea M. Moller-Vasquez, María Granados-Presa, Pamela Pennington, Norma Padilla, Sujata Balasubramanian, Lisa D. Auckland, Elsa Berganza, Estuardo Alvarado Liberato, Henry Esquivel, Ranferi Trampe, Louisa A. Messenger, Celia Cordón-Rosales, Gabriel L. Hamer, Sarah A. Hamer

**Affiliations:** 1 College of Veterinary Medicine & Biomedical Sciences, Texas A&M University, College Station, USA; 2 Centro de Estudios en Salud, Universidad del Valle de Guatemala, Guatemala City, Guatemala; 3 Sustainable Sciences Institute, Oakland, CA USA; 4 Departamento de Epidemiología de la Dirección Departamental de Redes de Servicios Integrados de Salud Jutiapa, Ministerio de Salud Pública y Asistencia Social, Jutiapa, Guatemala; 5 Sección de Vectores de Jutiapa de Redes de Servicios Integrados de Salud Jutiapa, Ministerio de Salud Pública y Asistencia Social, Jutiapa, Guatemala; 6 Parasitology and Vector Biology Laboratory (PARAVEC Lab), School of Public Health, University of Nevada, Las Vegas, NV USA; 7 Department of Environmental and Global Health, University of Nevada Las Vegas, Las Vegas, NV USA; 8 Department of Entomology, Texas A&M University, College Station, USA

**Keywords:** Triatomines, Follow-up, Chagas disease, Treatment, Persistent, Acute cases

## Abstract

**Background:**

*Trypanosoma cruzi*, the causative agent of Chagas disease, is primarily transmitted by triatomine insects, including *Triatoma dimidiata*. In Central America, vector control programs have significantly reduced transmission; however, certain regions, such as Comapa municipality, department of Jutiapa, Guatemala, continue to experience persistent *T. dimidiata* infestation. This study presents a 10-year follow-up assessment of triatomine infestation, *T. cruzi* infection and acute Chagas disease cases after an eco-bio-social intervention.

**Methods:**

Between June and August 2022, entomological surveys were conducted in four communities of Comapa municipality. Seventy-six households were systematically searched for triatomines using the one-person hour method. Triatomines were collected and processed for *T. cruzi* detection using real-time PCR (qPCR), and blood meal analysis was performed to assess host feeding patterns. Dog samples and environmental DNA from household surfaces were also processed for *T. cruzi* detection. Additionally, surveillance for acute Chagas disease cases was carried out in collaboration with the Ministry of Health.

**Results:**

Persistent household infestation by *T. dimidiata* was observed across all four communities, with infestation rates ranging from 17% to 38% and colonization levels ranging from 9% to 29%. The mean household triatomine density remained low, suggesting a possible reduction in transmission risk. A total of 86 triatomines were collected, of which 26% tested positive for *T. cruzi* (all TcI strain). Amplicon deep-sequencing analysis of the blood meals from triatomines identified seven vertebrate species and one insect family as hosts upon which triatomines had previously fed, with chickens and dogs being the most common blood meal sources (occurring in 85% of triatomines). Of the 132 dogs processed, 22% were positive for *T. cruzi* (all TcI strain). Two acute Chagas disease cases in children were detected in the surveillance period, including one child in 2015 who remained seropositive in 2022, emphasizing the need for continued surveillance.

**Conclusions:**

Despite the multiple interventions that have been carried out for over a decade in Comapa municipality, *T. dimidiata* infestation remains high in the area, with sustained evidence of acute Chagas disease in humans, necessitating continued vector control efforts. The persistence of *T. cruzi* transmission among triatomines and dogs and the predominant role of chickens and dogs in supporting the vector population highlight the need for innovative control strategies, including those that target domestic animals, to mitigate Chagas disease risk.

**Graphical Abstract:**

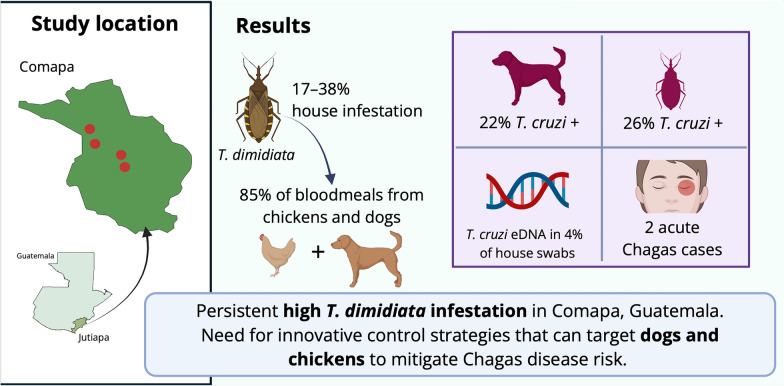

**Supplementary Information:**

The online version contains supplementary material available at 10.1186/s13071-025-06897-7.

## Background

Chagas disease, caused by the parasite *Trypanosoma cruzi* (Chagas, 1909), is a neglected tropical disease that infects around 7 million people worldwide, with most cases found in Latin America [1]. The disease is primarily transmitted by insects of the family Reduviidae [1]. Despite being considered one of the most neglected tropical diseases and having one of the highest disability-adjusted life years (DALYs) of any infectious disease in the Americas [2, 3], its control still heavily relies on traditional vector control strategies that focus on indoor residual spraying due to the lack of vaccines and drug therapies with lower toxicity and higher efficiency [4].

*Trypanosoma cruzi* infection has a 7- to 15-day incubation period after the initial infection, followed by an acute phase that lasts an average of 8 weeks, with diagnosis based on the detection of circulating parasites determined by blood Strout or blood smear examination. However, only 1–2% of infected individuals report symptoms at this stage, which include chagomas (inflammatory reactions at the inoculation site) and the Romaña sign (swelling of the eyelid), making the diagnosis of acute cases extremely rare [5, 6]. Chronic disease manifestations (including Chagas cardiomyopathy) may occur among a subset of infected individuals [7]. In Central America, vector control interventions based on indoor residual spraying (IRS) carried out during the first decade of 2000 have been reported to be responsible for reducing vectorial transmission by 94% [8]. However, there are still regions where persistent indoor infestation with *Triatoma dimidiata* (Latreille, 1811) remains high.

Comapa municipality (Comapa), department of Jutiapa, Guatemala is a region of sustained Chagas disease transmission, with indoor infestation of *T. dimidiata* of ≥ 15%, exceeding the 8% threshold considered necessary for sustainable disease control, even after multiple rounds of IRS [9]. In 2011, an entomological baseline survey of *T. dimidiata* confirmed high infestation indices across multiple communities of Comapa [9]. In 2012, a multi-stakeholder community-based intervention focusing on ecological, biological and sociological risk factors [9] was developed and successfully adopted by households for the management of rodents in the peridomicile to control Chagas disease [10, 11]. In addition, an intervention that focused on house improvement was developed in the same area [12]. Results from a 2015 seroprevalence study showed that seropositivity in school-age children had decreased from 9.2% before the mass insecticide applications to 1.6% over a decade after the mass insecticide applications were completed [13]. However, beyond understanding how an intervention might be adopted and implemented by community members [14], there is a need to evaluate the risk inhabitants might face in these communities with persistent *T. dimidiata* infestation and how other entomological indices, such as vector household density, could provide a more nuanced evaluation of household risk. In the present study, we report on triatomine infestation, *T. cruzi* infection and blood meal analysis results for four communities in Comapa, Jutiapa, as well as the level of infection in domestic dogs and occurrence of human disease.

## Methods

### Study sites and sample size

**Fig. 1:**
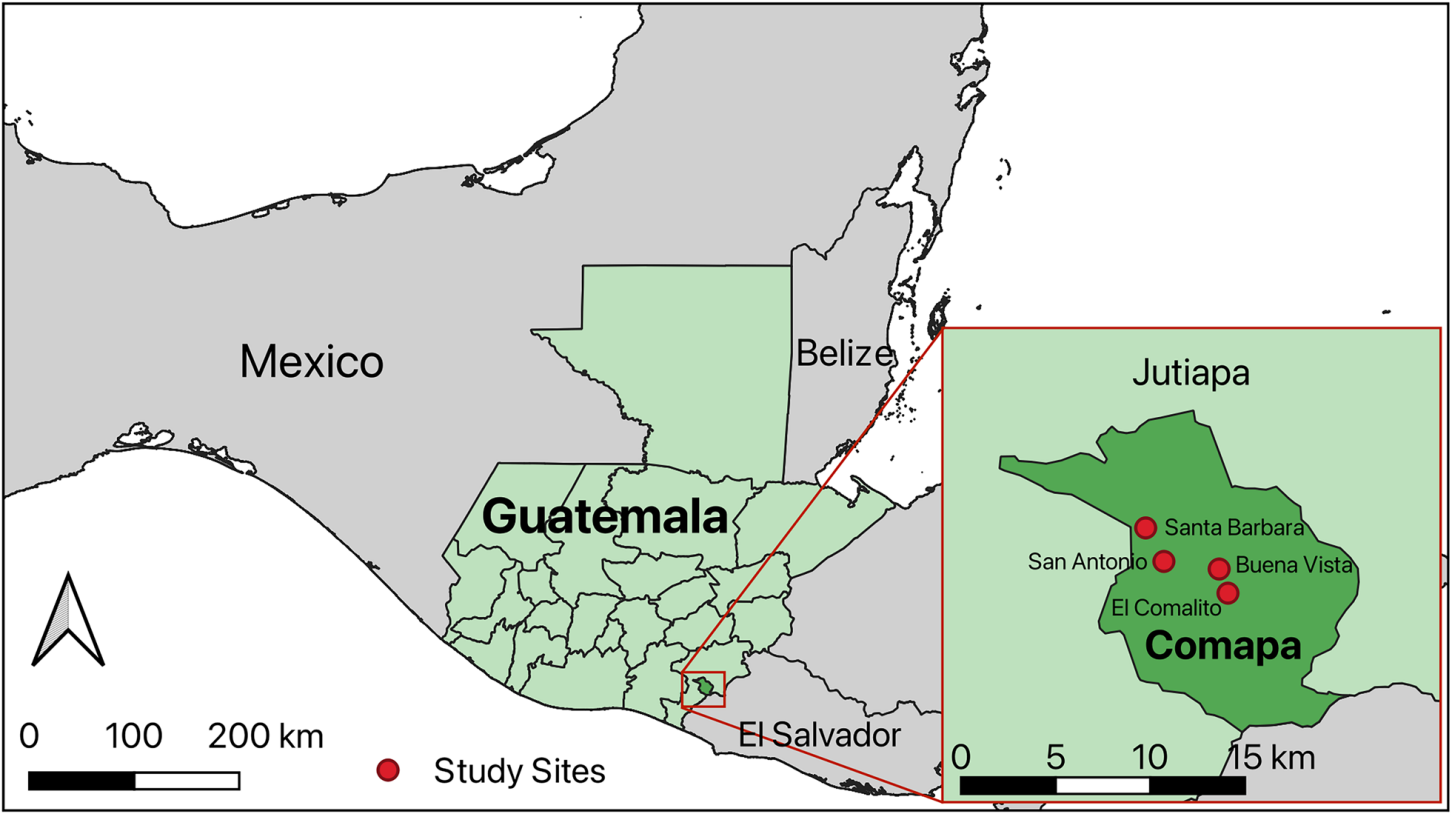
Triatomine sample sites in Comapa municipality, department of Jutiapa, Guatemala, 2022. Maps were generated using QGIS 3.30 using freely available administrative boundaries

The information on the study communities and prior interventions have been previously published [10]. Briefly, this research was conducted in the municipality of Comapa, department of Jutiapa, which is located in southeastern Guatemala (Fig. 1). The research was conducted in four randomly-selected communities of Comapa from June to August 2022 that had been included in a previous study: two communities had been control communities (Buena Vista [BV] and El Comalito [EC]) and two communities had been part of an intervention receiving education regarding risk factors and environmental management (San Antonio [SA] and Santa Barbara [SB]) [10]. In the previous study, we documented that > 15% of the households were infested with *T. dimidiata*, out of a total of 18 communities that took part in an eco-bio-social intervention for the control of Chagas disease [10, 11, 15]. As previously done, we intended to survey the same 24 households (as per the guidelines of the Guatemalan Ministry of Health (MoH) [16]) from the original selection of 2012 [10], to provide us with information regarding *T. dimidiata* infestation and *T. cruzi* infection rates 10 years after the intervention took place. Additionally, a member of the El Anonito community requested entomological surveillance help in her household (not part of the historical studies) where many triatomines occurred; the results for this household are presented separately in Additional file 1: Result S1.

### Entomological surveillance

The search for triatomines was conducted by our research team alongside MoH personnel following the one person-hour methodology [16]. The highly-trained MoH personnel thoroughly searched all premises of each participating community, the outside walls and peridomicile structures for triatomines. Search time could be extended depending on the number of rooms, items required to be moved and/or whether triatomines found. Triatomines were classified by developmental stage, feeding status, sex and species. All specimens were stored in 70% ethanol, and samples were later moved into RNAlater® (Invitrogen, Thermo Fisher Scientific, Waltham, MA, USA) and stored at − 80 °C until further processing for *T. cruzi* infection and blood meal analysis. Triatomines collected in the additional house from El Anonito were kept alive and taken to the laboratory at Universidad del Valle de Guatemala (UVG). Adult triatomines from El Anonito were not processed since they were used to establish a *T. dimidiata* laboratory colony; all instars were processed. Three entomological indicators were used to describe data: (i) infestation (% of households with triatomines); (ii) colonization (% of households with triatomine instars); and (iii) vector density (average of triatomines found in a household).

### Environmental DNA

Environmental sampling to detect environmental DNA (eDNA) of *T. cruzi* [17] was conducted using two polyester swabs, which were wiped over an approximate 60 × 60-cm area of the wall and floor adjacent to the bed in each household. Each surface was swabbed for approximately 15 s, and swabs were subsequently placed into a collection tube containing approximately 1 ml of RNAlater®. The samples were then frozen until further processing. DNA was extracted from 200 µl of solution using the Omega E.Z.N.A. Tissue DNA Kit (Omega Bio-tek Inc, Norcross, GA, USA), following the manufacturer’s protocol with a modification to the elution step: instead of a single elution step, we used a two-step elution in which 25 µl of elution buffer was added, followed by centrifugation, then an additional 25 µl was added before a second centrifugation, resulting in a final elution volume of 50 µl. Quantitative real-time PCR (qPCR) was performed to detect the presence of *T. cruzi* DNA as described in the following sections.

### Triatomine processing

All specimens were handled under biosafety level 2 hoods, with pictures taken and length measurements recorded. We dissected the body and separated the hindgut and remaining body into separate tubes for instars 4 and 5, and adults. This procedure involved first dipping the specimen in sterilized water for 10 s and then in 50% bleach for 15 s, followed by cutting the connexivum to remove the guts. Records on the feeding score (1–5) of each specimen were kept as follows: 1, no blood, no guts; 2, guts found, no feeding; 3, small traces of feeding; 4, evidence of feeding, but limited; and 5, evidence of feeding, engorged. Instars 3 and below were scored using a less resolved scale of fed, slightly fed or unfed.

We used the MagMax-96 DNA Multi-Sample Extraction Kit (Applied Biosystems, Thermo Fisher Scientific, Waltham, MA, USA) for the extraction of total nucleic acid from the hindgut of individual triatomines. If the tissue sample was > 10 mg, the volume of proteinase K would be doubled. The samples were left overnight at 55 °C in a shaker to achieve lysis, following which they were further processed following the procedure described previously [18]. The DNA was extracted in batches of 12 samples, consisting of 11 experimental samples and one extraction negative control containing only reagents. This extraction control was included in the subsequent PCR assay alongside the experimental samples to monitor for potential contamination. Each PCR run also included a non-template control to further confirm the absence of contamination during amplification.

### Dog sampling and processing

Dogs owned by household residents were first restrained and then fitted with a muzzle. The venipuncture site was disinfected with 70% ethanol and up to 5 ml of blood was collected into EDTA tubes, which were initially stored at 4 °C, followed by centrifugation, with the resulting serum and blood clot stored at − 80 °C. All personnel involved in dog handling and sample collection were vaccinated against rabies. Data including sex, age and breed were recorded for each dog. Data on the ectoparasites collected from these dogs have already been presented and analyzed in a separate published study [19].

### *Trypanosoma cruzi *detection and strain genetic characterization

qPCR was performed to detect the presence of *T. cruzi* in all samples (DNA from house swabs, triatomines and dog blood) using a Stratagene MxPro3000 qPCR system (Agilent Technologies, Santa Clara, CA, USA). All samples were processed using 3 µl of DNA, 0.3 μl of each primer (Cruzi 1: 5′-ASTCGGCTGATCGTTTTCGA-3; Cruzi 2: 5′-AATTCCTCCAAGCAGCGGATA-3; Cruzi 3: 5′-Fam-CACACACTGGACACCAA-NFQ-MGB-3) [20], 0.45 μM of probe and a mix of PCR FailSafe enzymes with PreMix E (Agilent Technologies). The amplification target was a 166-bp region of repetitive microsatellite nuclear DNA detected using TaqMan probes. Negative controls were DNA-free water, and positive controls were DNA recovered from *T. cruzi*-positive* Macaca fascicularis *(crab-eating macaque). Dog blood samples were additionally processed for antibody detection of *T. cruzi* with the off-label use of the Chagas Stat-Pak Assay (Chembio Diagnostic Systems, Medford, NY, USA), a rapid serological test, following the manufacturer’s procedures [21].

All samples that tested qPCR-positive for *T. cruzi* were subjected to genetic characterization of the parasite using a reverse transcription (RT)-qPCR of the SL-IR gene of *T. cruzi* with differential probes for each DTU [22, 23]. The protocol used a multiplex PCR kit (Qiagen, Hilden, Germany). Samples were processed at a final volume of 18 µl by reaction with positive controls for the discrete typing units (DTUs) [23]. Samples without a DTU identification were rerun at a 1:10 concentration, and if no positive result was obtained, they were left as untypable.

**Table 1:** Household triatomine indices for *Triatoma dimidiata* collections and *Trypanosoma cruzi* infection in four communities of Comapa municipality, department of Jutiapa, Guatamala

Variable	Overall	Community			
		BV^a^	EC^a^	SA^b^	SB^b^
Households	*N* = 76	*N* = 24	*N* = 5	*N* = 23	*N* = 24
*Infestation*					
Overall	20 (26%)	9 (38%)	1 (20%)	4 (17%)	6 (25%)
Domicile	14	7	0	4	3
Peridomicile	7	2	1	1	3
*Colonization*					
Overall	14 (18%)	7 (29%)	1 (20%)	2 (9%)	4 (17%)
Domicile	8	5	0	2	1
Peridomicile	6	2	1	0	3
*Life stage*	*N* = 86	*N* = 53	*N* = 3	*N* = 21	*N* = 9
1st instar	9 (10%)	6 (11%)	0 (0%)	3 (14%)	0 (0%)
2nd instar	25 (29%)	17 (32%)	2 (67%)	4 (19%)	2 (22%)
3rd instar	16 (19%)	7 (13%)	0 (0%)	7 (33%)	2 (22%)
4th instar	1 (1.2%)	1 (1.9%)	0 (0%)	0 (0%)	0 (0%)
5th instar	14 (16%)	9 (17%)	1 (33%)	1 (4.8%)	3 (33%)
Female	9 (10%)	6 (11%)	0 (0%)	3 (14%)	0 (0%)
Male	12 (14%)	7 (13%)	0 (0%)	3 (14%)	2 (22%)
*Location*					
Domicile	62 (72%)	37 (70%)	0 (0%)	20 (95%)	5 (56%)
Peridomicile	24 (28%)	16 (30%)	3 (100%)	1 (4.8%)	4 (44%)
*Trypanosoma cruzi*					
Negative	64 (74%)	39 (74%)	0 (0%)	19 (90%)	6 (67%)
Positive	22 (26%)	14 (26%)	3 (100%)	2 (9.5%)	3 (33%)
*Strain*					
Tc1	20 (91%)	12 (86%)	3 (100%)	2 (100%)	3 (100%)
Untypable	2 (9.1%)	2 (14%)	0 (0%)	0 (0%)	0 (0%)

*BV* Buena Vista,* EC* El Comalito,* SA* San Antonio,* SB* Santa Barbara

^a^Historical control community

^b^Historical intervention community

### Triatomine blood meal analysis

Blood meal analysis was based on deep sequencing of amplicons generated for a region of the vertebrate 12S ribosomal RNA (rRNA) mitochondrial locus amplified from the extracted DNA of triatomines [24–26]. Amplification of the 145-bp fragment was performed in duplicate for every sample. Forward and reverse primers included dual identical barcodes [27]. Samples were handled in biosafety cabinets and in separate pre- and post-PCR areas of the laboratory to minimize contamination. Samples with appropriately sized bands visible on gel electrophoresis were submitted to the Texas Institute for Genome Sciences (https://genomics.tamu.edu) for library preparation using xGen™ ssDNA and the Low Input DNA Library Prep Kit (Integrated DNA Technologies, Coralville, IA, USA) and sequencing on the Illumina NextSeq 2000 (Illumina, San Diego, CA, USA). Barcodes were trimmed and demultiplexed using Cutadapt 5.0 [28]. Sequences were imported into Qiime2 [29] and primers were trimmed, sequences were merged and denoised at minimum length of 100 bp. Representative sequences were matched to taxa using NCBI BLAST (National Center for Biotechnology Information, US National Library of Medicine) with MegaBLAST with the GenBank database (https://www.ncbi.nlm.nih.gov/genbank/) [30]. Only host species appearing in both replicates and showing > 500 reads were retained. Species-level identification of hosts was accepted at 99–100% identity to the database match. Lower percent identity scores were categorized at a genus level. If multiple matches were found with the same percentage identity, the host was identified up to the lowest common taxon.

### Acute Chagas disease surveillance

Since the time of our initial intervention project [9], the MoH established an active acute Chagas disease surveillance project between March 2013 and June 2015. All suspected acute Chagas cases (any person with Romaña sign or with chagoma) detected by community members, MoH personnel or UVG were referred to the epidemiology unit of Jutiapa. Households with a suspected acute case were visited by vector control and laboratory personnel from the MoH. The household was then searched for triatomines using the hour person method, and a 5 ml peripheral blood sample was collected from all children in the household for parasitological diagnosis using the Strout (< 7 years of age) technique [31]. Collected triatomines were transported alive to UVG laboratories for microscopic observation of *T. cruzi.* The identity of parasites observed in samples from children was confirmed by PCR and used to develop an in-house immunofluorescence assay panel [32]. Triatomines that arrived alive had their hindgut punctured, and then 100 µl of phosphate-buffered saline solution was used to wash the punctured area to extract a diluted portion of the fecal matter. *Trypanosoma cruzi* identity was confirmed by phase contrast microscopy at 600× magnification. Children positive for *T. cruzi* were tracked during November 2022 to follow-up on their Chagasic status.

### Statistical analysis

Initial descriptive analyses were performed for data exploration, and normality was evaluated using the Shapiro–Wilk normality test. Overall entomological comparisons between domicile and peridomicile household infestation and colonization were analyzed using a Pearson’s Chi-square test with Yate’s continuity correction, with *p*-value of < 0.05 indicating significance. Triatomine abundance between domicile and peridomicile by community was analyzed using the Wilcoxon Rank Sum test. All analyses and graphs were generated with R 4.4.1 [33]. Due to the small sample size, we avoided comparing data from El Anonito in the analysis. Maps were generated using QGIS 3.30 using freely available administrative boundaries.

## Results

### High infestation and colonization levels of* Triatoma dimidiata*

**Fig. 2:**
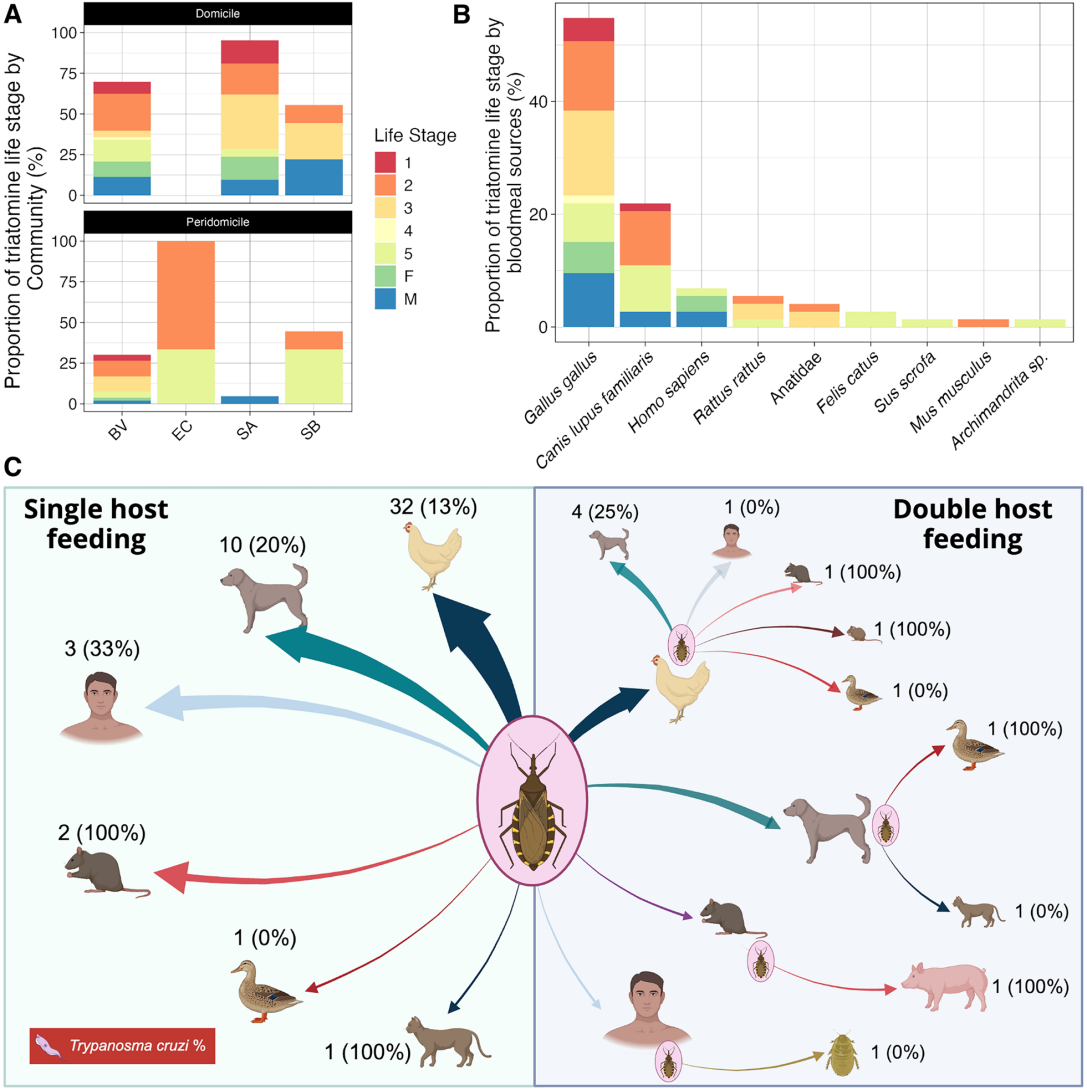
*Triatoma dimidiata* blood meal analysis in Comapa municipality, department of Jutiapa, Guatemala, 2022. **A** Relative abundance of domestic and peridomestic triatomine collections by life stages (1–5, first to fifth instar; F, female; M, male) and communities (BV, Buena Vista; EC, El Comalito; SA, San Antonio; SB, Santa Barbara). **B** Relative abundance of host feeding sources of triatomines by life stages. **C** Blood meal source for single and double host feeding, with percentage of positive *Trypanosoma cruzi* blood meals. The size of the arrow is proportional to the number of observed blood meals from a given host. Figure 2c was created in BioRender (J Juarez, 2025: https://BioRender.com/havdph3)

In the four communities, follow-up surveys were completed in 24 houses in both Buena Vista and San Antonio, 23 houses in Santa Barbara and five houses in El Comalito, for a total of 76 households (Table 1). We observed a high infestation level with *T. dimidiata* that ranged from 20% to 38% in the control communities (BV and EC) and from 17% to 25% in the intervention communities (SA and SB). Additionally, the colonization (presence of nymphal stages) of households ranged from 20% to 29% in the control communities and from 9% to 14% in the intervention communities. We did not observe any statistical difference between domicile and peridomicile household infestation and colonization. A total of 86 *T. dimidiata* specimens (65 instars, 21 adults) were collected across all four communities. The highest abundance of total triatomines (53 specimens: 40 instars, 13 adults) was recorded at BV (control community), where the mean density per household was 2.21 (standard deviation [SD] 3.99) triatomines. At SA (intervention community), a total of 21 triatomines (15 instars, 6 adults) were collected, with a mean density of 0.91 (SD 3.54). At SB (intervention community) and EC (control community), we recorded a total of nine triatomines (7 instars, 2 adults), with a mean density of 0.37 (SD 0.77), and a total of three triatomines (3 instars, 0 adults), with a mean density of 0.6 (SD 1.34), respectively. We observed a wide distribution of instars and adults in both domicile and peridomicile environments (Fig. 2a). The male:female ratio of adult insects collected inside domiciles was 14:11. The proportion of instars collected in the domicile varied from 40% to 60% of the overall collections, with no statistical difference observed between collection sites.

### *Triatoma dimidiata* and infection of dogs with* T. cruzi* TcI strain

Of the 86 triatomines processed for *T. cruzi*, we detected *T. cruzi* DNA in 22 (26%), all of which came from 10 households. Six households in BV (control community) were infested with *T. cruzi*-infected triatomines, as well as two households in SA (intervention community) and one household each in EC (control community) and SB (intervention community). We observed positive immatures as early as the first instar (*n* = 1 in BV); subsequent life stages were also infected as follows: second instar (*n* = 7 positives), third instar (*n* = 2 positives), fifth instar (*n* = 5 positives) and adults (*n* = 7 positives: 3 females, 4 males). We did not observe any statistical difference in infection of triatomines between domicile and peridomicile specimens by life stage. The only *T. cruzi* DTU detected in the 20 specimens that were positively typed was TcI [23].

We sampled blood collected from a total of 132 dogs, of which 19 (14%) had blood that was qPCR-positive for *T. cruzi* and 21 (16%) were antibody-positive. When the results were combined, we observed that 28 (21%) dogs were positive by either or both methods. Of those qPCR-positive dogs, the DTU was determined for 11 samples, and all were found to be the TcI strain. Dogs as early as 2 month olds were found positive for *T. cruzi*.

### Detection of household* T. cruzi* eDNA

Six of the 145 swabs were positive for *T. cruzi*; these swabs were from five different households, with both the wall and floor swabs in one household positive for *T. cruzi*. We determined the DTU of two samples as TcI. The highest number of positive samples was recorded on the floors adjacent to the edge of the bed (*n* = 4), while only two positive samples were detected on the walls.

### Blood meal analysis of* T. dimidiata*

Of the 86 triatomines collected, 61 showed amplicons of the appropriate size after PCR for the vertebrate 12S rRNA gene. Seven vertebrate hosts were identified by species (*Canis lupus familiaris* (Linnaeus, 1758), *Felis catus* (Linnaeus, 1758), *Gallus gallus* (Linnaeus, 1758), *Homo sapiens* (Linnaeus, 1758), *Mus musculus* (Linnaeus, 1758), *Rattus rattus* (Linnaeus, 1758), *Sus scrofa* (Linnaeus, 1758)) and one by family (Anatidae) (Fig. 2b). Additionally, one sequence matched *Archimandrita* sp. (TD-3231-3), which is an invertebrate genus of cockroach; incidentally, many* Archimandrita* species were observed in sampling locations at the time of collections (see Additional file 1: Figure S1). Most triatomines (*n* = 49) had sequences for a single host species, and 12 showed two different hosts (Fig. 2c). The most frequent blood meal was *Gallus gallus* (32 one-host blood meals and 8 two-host blood meals), followed by *Canis lupis familiaris* (10 one-host and 6 two-host blood meals) and *Rattus rattus* (2 one-host and 2 two-host blood meals). Human blood meals were identified from three triatomines as the sole blood meal host (1 *T. cruzi*-positive) and from two other triatomines along with *Gallus gallus* and *Archimandrita* sp.

### The tip of the iceberg: acute Chagas disease

**Fig. 3:**
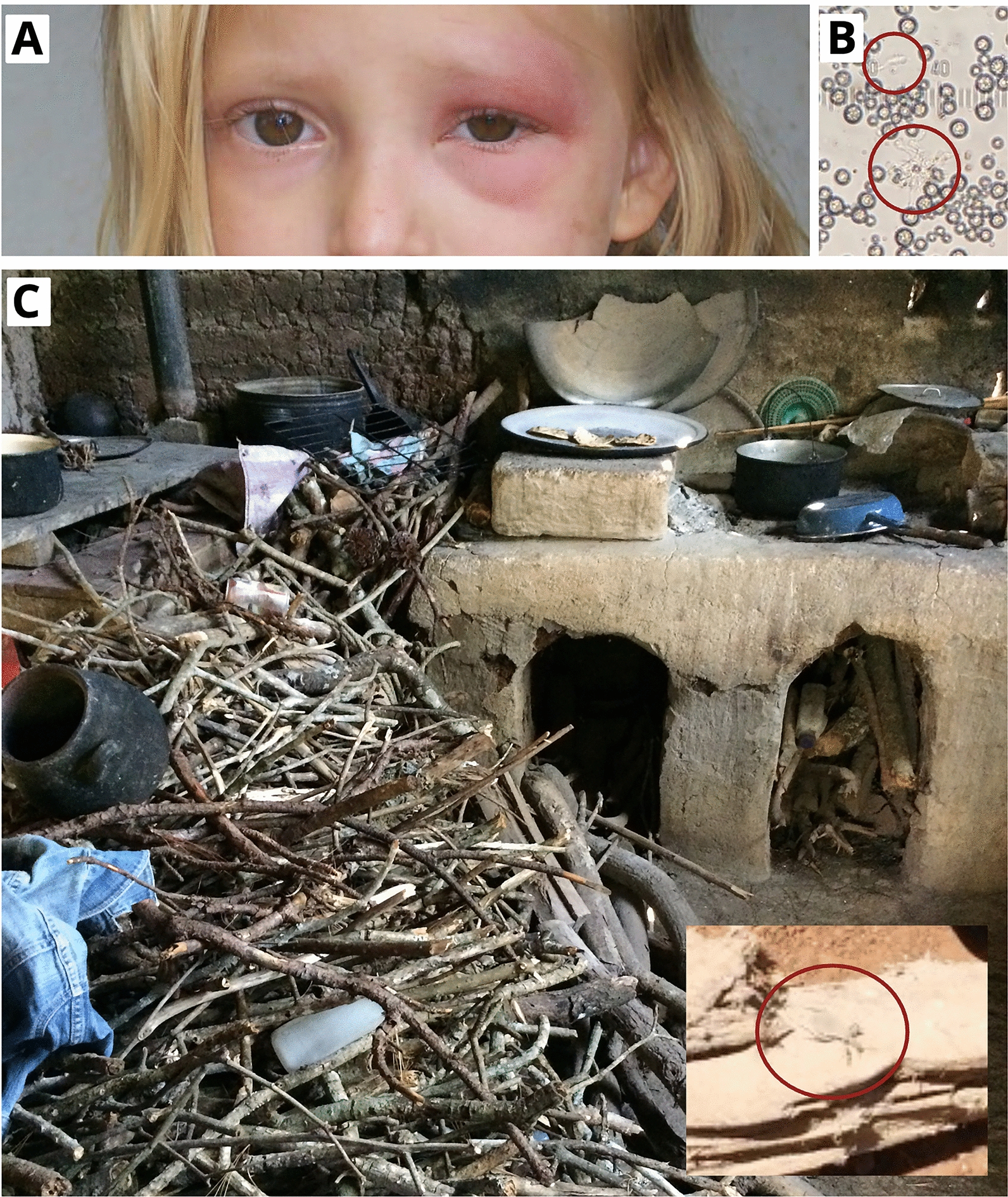
Acute case of Chagas disease in a child from Comapa municipality, department of Juatiapa, Guatemala in 2015. **A** Romaña sign characterized by eyelid swelling of the left eye in the 4-year-old girl from Santa Bárbara, Comapa, **B** *Trypanosoma cruzi-like* trypomastigote from the hindgut of a *Triatoma dimidiata* nymph. **C** Kitchen adjacent to the child’s bedroom, with inset showing a fourth instar nymph collected in the wood from the kitchen

In March 2014, a 6- to 10-year-old (y/o) boy from the community of San Juan, Comapa, was taken to the local health center because he presented Romaña’s sign and a 1-week-long fever. The child was discharged, and a community health promoter recognized the symptoms and alerted vector control personnel of a probable acute Chagas disease case. A Strout test confirmed acute *T. cruzi* infection, and entomological inspection of the household revealed a single female *T. dimidiata* in the child’s bedroom, which tested positive for *T. cruzi* by rectal puncture and microscopy. In April 2015, a 1- to 5-y/o girl from Santa Bárbara, Comapa, exhibited Romaña’s sign (Fig. 3a) and mild fever. A health educator trained in Chagas disease identification reported the case to Jutiapa’s epidemiology unit. A household inspection revealed 25 *T. dimidiata* specimens in the kitchen (Fig. 3c) adjacent to the child’s bedroom. Thirteen specimens were transported alive to UVG, where three tested positive for *T. cruzi* (Fig. 3b). The child also tested positive for *T. cruzi* via Strout and hemoculture. The remaining children in the household were also tested via Strout and enzyme-linked immunosorbent assay (ELISA) with negative results. Both Chagas-positive children were treated with nifurtimox under the supervision of a certified MoH nurse, who followed the protocol for Chagas disease management from the Guatemalan MoH [31]. In November 2022, the MoH conducted a follow-up to assess the children’s Chagasic status. Unfortunately, only the girl was located, and her ELISA test remained positive, which could be due to insufficient time for antibody waning, or possible re-infection, as *T. dimidiata* remains prevalent in the region.

## Discussion

The Chagas disease vector control program in Guatemala has been extremely successful, with only a few pockets of persistent infestation remaining. One such pocket of persistent high infestation is in the southeastern region of Guatemala, which has shown refractoriness to multiple vector control activities and interventions over the years. Our results showed that high infestation levels remain in the four communities evaluated and, even more importantly, that infection rates with *T. cruzi* were widespread in all communities and in all life stages as early as first instars. Dogs as young as 2 months of age were also found to be *T. cruzi*-positive. *Trypanosoma cruzi* DTU TcI remains the sole DTU detected in both triatomines and dogs in the present study, consistent with the results of our prior study and suggesting no new DTU introductions [34]. Importantly, the follow-up of acute Chagasic children showcased that even after treatment, patient monitoring is critical to inform diagnosticians on how to proceed with the management of Chagas disease and how the efforts of treatment might be thwarted by the persistence of triatomines in the region.

The 10-year follow-up evaluation of *T. dimidiata* in these four communities of Comapa municipality, department of Jutiapa showed that persistent infestation and colonization remain high in the region. Despite multiple efforts to reduce infestation levels through both traditional and ecological interventions [11], which have been widely accepted by community members [14], infestation persists. In contrast, a 20-year post-evaluation of an eco-health intervention in the community of La Brea, located in another region of Jutiapa, demonstrated promising results [35], suggesting potential interruption of disease transmission with high community acceptance. However, similar approaches in Comapa have not yielded the same success observed in La Brea. Another factor that could affect the persistence of *T. dimidiata* in this region is insecticide resistance, as has been observed in other regions and in other triatomine species [36, 37]. However, IRS in Comapa municipality is applied by MoH personnel in a very focalized manner and infrequently, suggesting that insecticide resistance may not be a main driver of re-infestation. One alternative possibility is that *T. dimidiata* in this region is due to a bounce back of sylvatic populations dispersing to the domicile. This refractoriness to both traditional and ecological control strategies highlights the need for sustained vector surveillance and novel control efforts in this region, as complete interruption of vector-borne transmission may be unfeasible, similar to other areas in the Americas [38]. We also observed that the mean density of households with triatomines remained at < 1 in all but one community, comparable to values reported in the 2011 survey [13]. Interestingly, the values of 2011 were observed when communities had been recently intervened with focalized spraying, a result also seen by Hashimoto et al. [39]. The household density levels for both *Rhodnius prolixus* [40] and *Triatoma infestans* [41] are typically much higher than those for *T. dimidiata,* which may serve as a more precise indicator of *T. cruzi* transmission risk. Our findings suggest that even in communities with persistent high infestation and colonization, household vector density may remain low enough to reduce the risk of *T. cruzi* transmission. This is supported by the observed decline in seroprevalence among school-aged children over the years [13], despite not reaching the 8% infestation threshold thought to be needed for disease transmission interruption [42].

*Trypanosoma cruzi* infections were observed across all life stages of triatomines, as early as first instars. Our results show a comparable infection prevalence to that observed in the 2011 baseline when 31% of triatomines were positive for *T. cruzi* [9]. This infection prevalence has also been observed in other rural communities from the southern region of Mexico, where limited control measures have been applied since Chagas disease is not considered to be an endemic disease for that region [43]. Importantly, the infection prevalence remains below what is generally observed in South American countries where infection prevalence in triatomines can reach up to 68% [44, 45].

In our baseline study over a decade earlier in these communities, seroprevalence in 80 adult dogs was 37% [9], compared to 16% in the present study, although different diagnostic approaches add complexity to our assessment of change over time. We observed that one-third of all infected dogs were younger than 12 months. Given the limited known travel of these dogs, the data suggest active transmission in the household and, more importantly, that these dogs could serve as household reservoirs, ultimately posing a risk for continued human transmission.

Our eDNA testing showed that a simple swab from a house wall or floor may be processed to show evidence of the *T. cruzi* parasite, likely by picking up remnants of parasite from areas where infected triatomines have defecated. Given that swabbing a home requires no specialized training, such an approach could augment traditional methods of manual triatomine searching of homes. A mixture of traditional and more novel approaches should be considered to improve surveillance methods [17, 46], ultimately providing an in-depth understanding of the ecology of triatomine species.

In our previous study from 2011 in these rural Guatemalan communities, using conventional PCR we showed host feeding by triatomines was highest for *Gallus gallus* (64%), followed by humans (50%), dogs (17%), *Rattus rattus* (24%) and *Mus musculus* (21%) [9]. In the current study, we applied a more sensitive approach of blood meal metabarcoding and revealed a similar host community composition over a decade later. *Triatoma dimidiata* is an opportunistic feeder, and blood meals have been shown in different studies to include all classes of vertebrates [47–49]. The *T. dimidiata* specimens examined in this study revealed feeding on eight vertebrate hosts, with the majority of blood meals including *Gallus gallus* and/or *Canis lupus familiaris*, suggesting that chickens and dogs robustly support *T. dimidiata* in these environments. A total of five human blood meals were observed, of which three were solely human and two showed the presence of other hosts as well.

This predominance of feeding on chickens and dogs in this population of bugs that also feeds on humans underscores the need for targeted interventions that may include both *Gallus gallus* and *Canis lupus familiaris*. For example, chickens have been targeted with systemic insecticides to reduce mosquito populations that can transmit West Nile virus [50]. Dog infection rates and frequency of human blood meals in triatomines could in the future be used as outcome variables to analyze the impact of interventions in the short term.

Detecting acute Chagas disease remains a major challenge in vector-borne disease surveillance programs. The identification of cases relies on the direct ability of health care personnel in endemic areas to recognize the symptoms and physical manifestations of the infection [51]. Nonetheless, awareness at multiple levels of the healthcare system can significantly improve detection. In this specific scenario, health promoters played a key role in identifying multiple acute Chagas cases in remote villages of Guatemala. Since 2019, Comapa municipality has maintained a dedicated Chagas disease clinic for diagnosis, treatment, and follow-up. Improving awareness and detection of the disease, as well as laboratory diagnostics of acute cases, is critical to providing immediate treatment to patients, which has high efficacy [51]. However, seropositivity can persist long after successful parasite clearance, complicating treatment outcome evaluations. Studies have shown that in adults treated with nifurtimox, fewer than 60% achieved seronegative conversion even after 5 years [52], highlighting that seroreversion is a slow process rather than an indicator of treatment failure. Similarly, in pediatric cases, parasitological clearance confirmed by PCR occurred within 3 years, but conventional serology remained positive for most patients [53]. This shows the clear need to sustain vector control programs that rely on the use of both insecticide and non-insecticide-based approaches to reduce vector-human contact [10]. Given the prolonged time required for seroreversion, continued monitoring of vector populations and re-infection potential in endemic regions should remain a priority to ensure that achievements made through treatment are not undermined by persistent transmission cycles.

We acknowledge that community sample size in the current study hinders extrapolation to broader geographic regions and does not allow a robust test of the long-term impact of the historic eco-bio-social intervention from 2011. However, the present evaluation provides clear and robust information on the ecological patterns of *T. dimidiata* infestation, infection, and blood meal source in these communities. Additionally, contemporary molecular approaches, including the household eDNA sampling and blood meal metabarcoding, provide deeper insight into patterns of infestation and host sources for triatomines. Interestingly, this blood meal analytic procedure allowed us to capture presumed triatomine feeding on *Archimandrita* sp.; specimens of this large-bodied cockroach were observed in several households across the study sites (see Additional file: Figure S1), which showcases the opportunistic feeding nature of *T. dimidiata*.

## Conclusions

The persistence of *Triatoma dimidiata* infestation and *Trypanosoma cruzi* transmission in Comapa municipality, department of Jutiapa, underscores the complexity of Chagas disease control in endemic regions. Despite multiple intervention strategies over the past decade, infestation rates remain high, and *T. cruzi* infection continues to be detected in both vectors and domestic dogs. The detection of human blood meals in triatomines highlights an ongoing risk of transmission, reinforcing the need for innovative and sustainable vector control measures. Additionally, the follow-up of acute Chagas disease cases emphasizes the importance of long-term patient monitoring and strengthened diagnostic and treatment strategies. Our findings suggest that while conventional vector control methods have had some impact, more comprehensive and adaptive approaches—including ecological, community-based and integrated vector management strategies—are required to mitigate the risk of Chagas disease transmission in this region. Continued surveillance and collaboration with local stakeholders will be essential for developing effective and sustainable solutions for Chagas disease prevention and control.

## Supplementary Information

**Additional file 1: Results S1.** Results of entomological surveillance in a household in El Anonito community. **Figure S1:** *Archimandrita* sp. cockroach observed in sampling location at the time of triatomine collection.

## Data Availability

The data set is available in ZENODO: https://zenodo.org/records/14989960, Dryad: 10.5061/dryad.pzgmsbczg and R code can be found at: https://github.com/jgjuarez/Triatoma_2022
