# Supplementary material for: Triatoma dimidiata, domestic animals and acute Chagas disease: a 10-year follow-up after an eco-bio-social intervention
Source: Parasit Vectors. 2025 Jul 4;18:253. doi: 10.1186/s13071-025-06897-7 (PMC12228295; doi:10.1186/s13071-025-06897-7)
Supplement: Supplementary file 1 — Additional file 1: Results S1. Results of entomological surveillance in a household in El Anonito community. Figure S1: Archimandrita sp. cockroach observed in sampling location at the time of triatomine collection. [file 13071_2025_6897_MOESM1_ESM.docx]

**Additional file for the Manuscript:**

***Triatoma dimidiata,* domestic animals and acute Chagas disease: A 10 year follow-up after an eco-bio-social intervention**

Files included:

- Results S1
- Figure S1

**Results S1**

**El Anonito: *T. dimidiata* collection and infection with *T. cruzi* TcI strain**

The findings from a home in El Antonio are included as supplemental as this home was not part of the follow-up study. A total of 29 *T. dimidiata* specimens were collected in this household, with 28 nymphs and 1 adult. Specimens were collected in the peridomicile, in a storage room for firewood. All nymphs from 1^st^ (7), 2^nd^ (9) and 3^rd^ (3) instars were processed as described in the main manuscript. We collected one fourth instar nymph, 2 fifth instar nymphs and 1 adult male which were allowed to develop to use for colony establishment at UVG medical entomology laboratory. We detected 15 *T. cruzi* positive triatomines, of which 13 were determined as TcI strain. Infection was detected as early as 1^st^ instar, with 2^nd^, 3^rd^ and 4^th^ also being *T. cruzi* positive.

**El Anonito: Bloodmeal analysis**

We detected a positive bloodmeal from 14 of the 25 nymphs stored in the field. The most frequent meal was *Rattus rattus* (12 one-host bloodmeals and 2 two-host bloodmeals) followed by *Gallus gallus* (1 one-host and 1 two-host bloodmeals). We detected a 2^nd^ instar with a positive human bloodmeal with a dual feeding with *Rattus rattus*, this specimen was also positive for *T. cruzi*.

**
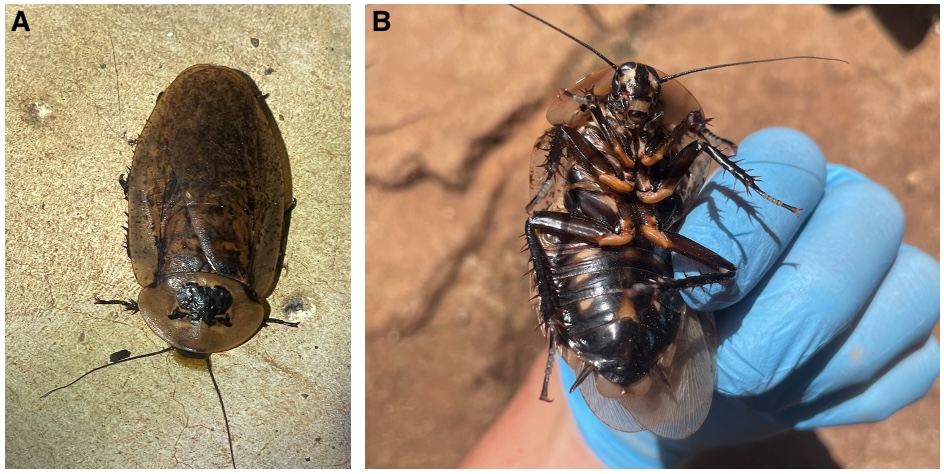
**

**Figure S1.** *Archimandrita* sp. cockroach found in a household of Comapa, Juatiapa were *T. dimidiata* was collected. A) Dorsal view. B) Ventral view. *Archimandrita* sp. was detected in the bloodmeal analysis procedure of triatomines suggesting triatomine feeding on hemolymph of this species.
